# The prevalence and risk factors for phantom limb pain: a cross-sectional survey

**DOI:** 10.1186/s12883-024-03547-w

**Published:** 2024-02-06

**Authors:** Katleho Limakatso, F. Ndhlovu, A. Usenbo, S. Rayamajhi, C. Kloppers, R. Parker

**Affiliations:** 1 Pain Management Unit, Department of Anaesthesia and Perioperative Medicine, Neuroscience Institute, University of Cape Town, Cape Town, South Africa; 2 Neural Prosthetics and Pain Research Unit, Bionics Institute, 384-388 Albert St, East Melbourne, East Melbourne, 3002 Australia; 3 Department of Anaesthesiology, Nelson Mandela Academic Hospital, Mthatha, South Africa; 4 Department of Acute Care Surgery, Groote Schuur Hospital, Cape Town, South Africa; 5 Pain Management Unit, Department of Anaesthesia and Perioperative Medicine, D23 Groote Schuur Hospital, Faculty of Health Sciences, Anzio Rd, Observatory, Cape Town, 7925 South Africa

**Keywords:** Phantom limb pain, Prevalence, Risk factors, Limb amputations, Africa

## Abstract

**Background:**

We previously performed a systematic review and meta-analysis which revealed a Phantom Limb Pain (PLP) prevalence estimate of 64% [95% CI: 60.01–68.1]. The prevalence estimates varied significantly between developed and developing countries. Remarkably, there is limited evidence on the prevalence of PLP and associated risk factors in African populations.

**Methods:**

Adults who had undergone limb amputations between January 2018 and October 2022 were recruited from healthcare facilities in the Western and Eastern Cape Provinces. We excluded individuals with auditory or speech impairments that hindered clear communication via telephone. Data on the prevalence and risk factors for PLP were collected telephonically from consenting and eligible participants. The prevalence of PLP was expressed as a percentage with a 95% confidence interval. The associations between PLP and risk factors for PLP were tested using univariate and multivariable logistic regression analyses. The strength of association was calculated using the Odds Ratio where association was confirmed.

**Results:**

The overall PLP prevalence was 71.73% [95% CI: 65.45–77.46]. Persistent pre-operative pain, residual limb pain, and non-painful phantom limb sensations were identified as risk factors for PLP.

**Conclusion:**

This study revealed a high prevalence of PLP. The use of effective treatments targeting pre-amputation pain may yield more effective and targeted pre-amputation care, leading to improved quality of life after amputation.

**Supplementary Information:**

The online version contains supplementary material available at 10.1186/s12883-024-03547-w.

## Significance

This study reports on the prevalence of, and risk factors for, Phantom Limb Pain in a sample of South African participants with amputations. The results of this study may help to strengthen efforts to optimise recovery from amputation surgery and to reduce suffering and disability in people with amputations. The knowledge of the risk factors for PLP in this population may yield more effective and targeted pre- and post-amputation care, leading to reduced healthcare utilisation and improved quality of life.

**Supplementary Information:**

The online version contains supplementary material available at 10.1186/s12883-024-03547-w.

## Introduction

The incidence of major limb amputations is high. Approximately 356 million limb amputations are conducted globally every year [1]. The sharp increase in the number of amputations in the past 10 years, primarily due to uncontrolled diabetes, has contributed to an increase in the prevalence of post-amputation complications including PLP [2].

Phantom limb pain is a common complication in people who have undergone limb amputations. A recent meta-analysis showed that roughly 64% [95% CI: 60.0–68.1] of people with amputations worldwide are affected by PLP [3]. This high and important statistic underscores the necessity of comprehensively elucidating the mechanisms that underlie PLP for effective management.

The current evidence points to the spontaneous nociceptive firing of the severed nerve and maladaptive cortical reorganisation as important drivers of PLP [4, 5]. It is hypothesised that spontaneous nociceptive firing of the afferent nerve is primarily responsible for the onset of acute PLP, and that maladaptive cortical reorganisation is involved in the maintenance of pain in the long term [5]. A recent systematic review suggests PLP is predicted by undergoing amputation due to uncontrolled diabetes, not receiving pre-amputation counselling, and having persistent pre-amputation pain, residual limb pain, and non-painful phantom sensations [3].

Remarkably, there is limited evidence on the prevalence of PLP and associated risk factors in African populations [3]. Therefore, the burden of pain and potential targets for treatment in this patient group are not clearly understood. In addition, it may be inappropriate to extrapolate the current evidence to the African context because of disparities in patient demographics and socioeconomic determinants of pain between developing and developed countries [6, 7]. The differences in these key determinants of pain highlight the need for an investigation focusing on the African population. Therefore, we conceived this study to explore the prevalence of PLP and associated risk factors in South African people with Lower Limb Amputations (LLAs).

## Methods

This study was designed using the STROBE checklist for observational studies (Supplementary file 1) [8]. Ethical approval for this study was granted by the Faculty of Health Sciences Human Research Ethics Committee of the University of Cape Town [ref: 066/2020].

### Study design

We conducted a cross-sectional study with a convenience sample of people with LLAs.

### Research setting

The study was conducted at three tertiary healthcare facilities based in the Western Cape and Eastern Cape provinces. A large proportion of individuals residing in these provinces come from households with low to middle income and present with comorbidities (e.g. diabetes) that often result in complications indicating a limb amputation [2].

### Recruitment

We recruited the participants for this study from a pre-existing ethics-approved database held by the acute care surgical unit at Groote Schuur Hospital [Acute care surgery online database- HREC 020/2018]. Additional participants were identified from the registries held in three other tertiary facilities in the Eastern Cape province. These registries include individuals who have undergone limb amputations and provided informed consent to be contacted for research purposes. The names and contact details of individuals who had undergone limb amputations between January 2018 and October 2022 were retrieved and entered into an Excel spreadsheet in date order from the first surgery performed in 2018 to the last surgery performed in October 2022. Individuals were contacted telephonically starting with the first allocated number to inform them (in the language they comprehend best) about the study and to invite participation. Those who verbally consented to participation were screened for eligibility against the inclusion/exclusion criteria. We included adults (≥ 18 years) who had undergone surgical or traumatic LLAs between January 2018 and October 2022 and were able to speak English, isiXhosa, or Afrikaans. These are native languages in the Eastern and Western Cape provinces of South Africa, where this study was conducted. Participants with auditory or speech impairments that hindered clear communication via telephone were excluded. In addition, we excluded patients who could not be reached via telephone after being contacted for three consecutive days at different times.

### Sample size determination

The sample size was calculated using the formula [$n=\frac{{Z}^{2} P(1-P)}{{d}^{2}}$] developed by Daniel [9] for calculating a sample size in prevalence studies. “Z” represents the Z statistic for a level of confidence, “P” the expected prevalence, and “d” the precision of the 95% confidence interval. Using the Z statistic of 1.96 (for 95% CI), an expected prevalence of 64% (based on the pooled prevalence estimate in our meta-analysis), and a precision of 0.053 (based on the 95% CI of the pooled prevalence estimate in our meta-analysis), a sample of 316 participants was required for a 95% confidence level.

### Outcomes

The primary outcome was PLP assessed using the pain severity scale of the Brief Pain Inventory (BPI) [10]. The first question of the BPI requires individuals to indicate (by circling ‘yes’ or ‘no’) whether they experienced pain during the week preceding data collection. The second part consists of four questions that ask participants to rate the severity of their worst, average, least, and current pain on a 0 to 10 scale, with 0 representing ‘no pain’ and 10 representing ‘pain as bad as you can imagine’. The pain severity score is calculated as the mean of the four ratings (out of 10). The BPI has been psychometrically validated in the three languages used in this study [11]. The secondary outcome was PLP risk factors assessed using a pre-piloted customised tool (Supplementary file 2 A). The tool evaluated pre-operative, peri-operative, and post-operative risk factors that consistently showed a positive correlation with PLP in our systematic review [3]. The participants indicated whether they were exposed to any of the risk factors by ticking either ‘Yes’ or ‘No’.

### Piloting

The random selection process and data collection forms were piloted in a small-scale study of 10 participants. The piloting process was conducted to determine the time to complete data collection with each participant and the feasibility of completing the questionnaires via telephone. The pilot data were used to pre-test and adapt the planned data import, tidying, and analysis processes. The data collection process was revised to separately collect data for each amputated limb instead of focusing only on the most recently amputated limb in people with double amputations. As a result, the data analysis plan was revised in a way that PLP prevalence was calculated by dividing the number of PLP cases by the number of amputation cases.

### Procedure

A trained healthcare professional fluent in the English, isiXhosa, and Afrikaans languages contacted the patients telephonically for data collection. The patient demographics questionnaire was used to collect demographic data including the number of amputated limbs per participant. In a case where the participant had multiple amputated limbs, outcome data on each amputated limb were collected. The participants were asked if they had experienced PLP in the previous week. Only those who responded with a ‘’yes” were asked to complete the pain severity scale of the BPI and provide details of their pain characteristics, including the nature, duration and frequency of pain episodes, specific to the affected limb (Supplementary file 2B). Participants with and without PLP were then screened for risk factors associated with PLP.

### Statistical analysis

Data were analysed using ‘R’ version 4.2.2 (Supplementary file 3). The prevalence of PLP was calculated by dividing the number of PLP cases by the number of amputation cases. For example, if the participant had two amputated limbs but had PLP in one of them, we recorded this as one PLP case and two amputation cases. The overall prevalence of PLP was expressed as a percentage with a 95% confidence interval. The association between risk factors and PLP was tested using univariate logistic regression analyses. Covariates associated with PLP at this stage were entered into the multivariable logistic regression model to examine the adjusted effects of the variables on the association between covariates and PLP [12]. We excluded interrelated independent variables (e.g., pre-amputation depression and post-amputation depression) to increase the robustness of the multivariable model. The associations between covariates and PLP were reported as Odds Ratio (OR) with a 95% confidence interval [13]. The Hosmer-Lemeshow test was conducted to assess the goodness of fit for the logistic regression model [14]. The median and Inter-Quartile Range (IQR) were used to analyse numerical baseline data. Characteristics of PLP were reported descriptively. Statistical significance was set at *p* < 0.05 for all analyses.

## Results

**Fig. 1:**
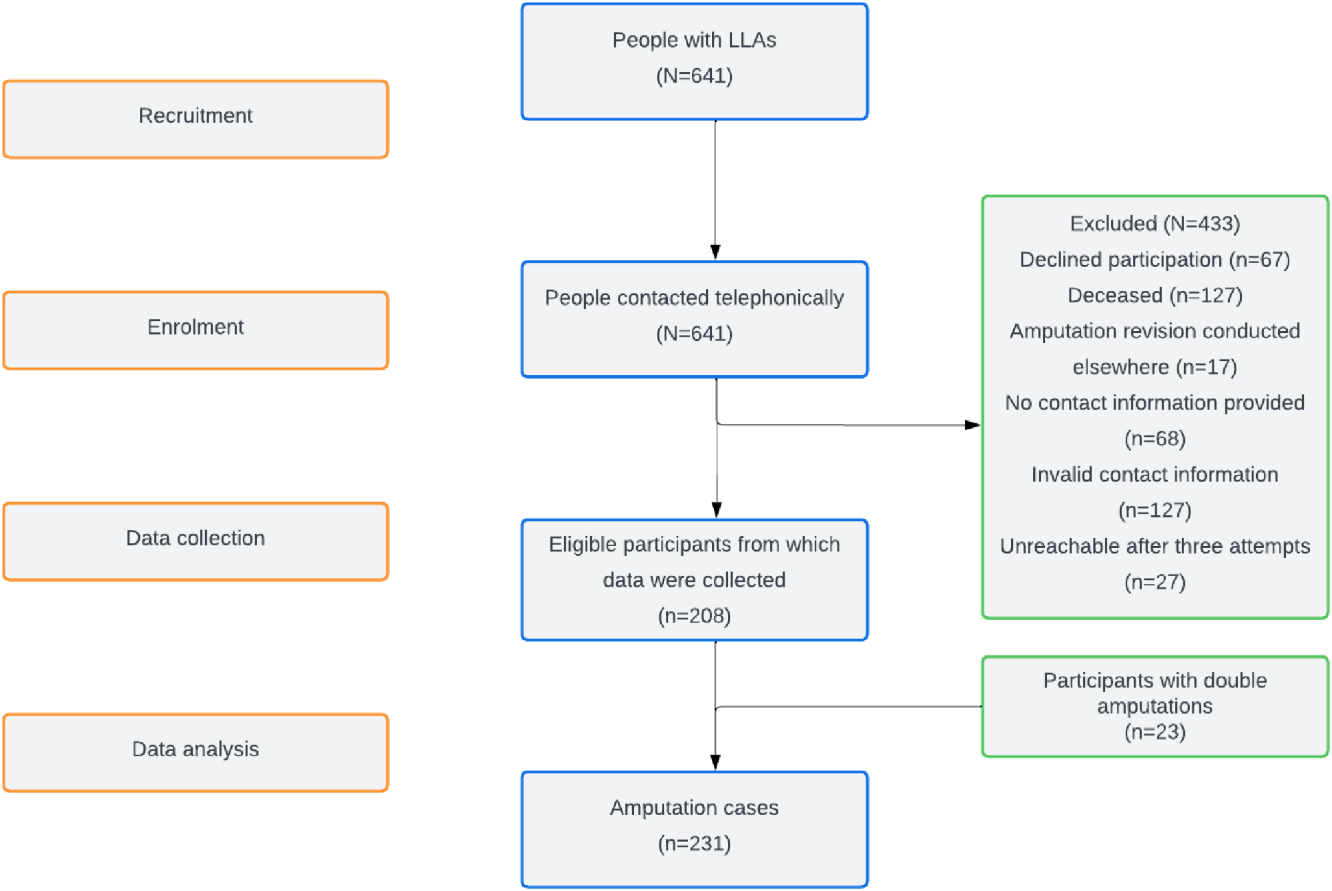
The STROBE flow diagram illustrating the recruitment, data collection, and data analysis processes

The results of the recruitment process are illustrated in Fig. 1.

The study included 208 participants [male (n = 133); female (n = 75)] with a mean (SD) age of 57.8 (12.8) (Table 1). Because 23 participants had undergone double amputations, the analysis was performed on a total of 231 cases. All the participants had LLAs, with most having amputations above the knee (57%). The participants had undergone amputation surgery approximately 10 months before recruitment. The common indications for amputation were complications due to uncontrolled diabetes, infection, limb ischaemia, and cancer. The terms that were commonly used to describe the PLP were sharp, burning, and shooting (Table 2).

**Table 1:** A summary of the demographic characteristics of the participants (n = 106)

Variable	Measure
Number of participants [n (%)]	
All the participants	208 (100)
Male	133 (64)
Female	75 (36)
Age [mean (SD)]	
All the participants	57.8 (12.8)
Male	59.9 (10.9)
Female	56.5 (13.6)
Level of amputation [n (%)]	
Above knee amputation	132 (57)
Below knee amputation	99 (43)
Months since amputation [mean (SD)]	
All the participants	9.76 (9.32)
Indications for amputation [n (%)]	
Diabetic complications	139 (60)
Limb ischaemia	68 (30)
Infection	16 (7)
Trauma	5 (2)
Cancer	3 (1)
Employment status [n (%)]	
Employed	14 (6.7)
Unemployed	194 (93.3)

**Table 2:** The symptoms of PLP and number of people experiencing them

PLP symptoms	People experiencing PLP symptoms [n (%)]^*^
Sharp	22 (33.8)
Shooting	16 (24.6)
Burning	15 (23.1)
Cramping	5 (7.7)
Dull	5 (7.7)
Itchiness	3 (4.6)
Stabbing	3 (4.6)
Shocking	3 (4.6)
Pinching	2 (3.1)
Pins and needles	2 (3.1)
Numbness	2 (3.1)
Piercing	1 (1.5)
Throbbing	1 (1.5)

*The percentage does not add up to 100% because some participants reported more than one symptom

### Prevalence and characteristics of PLP

The prevalence of PLP during the week preceding data collection was 71.73% [95% CI: 65.45–77.46]. The participants who reported pain experienced a mean (SD) of 3.88 (2.34) PLP episodes per week, with a mean (SD) pain severity score of 2.19 (1.81). The post-hoc analysis of ‘worst pain’ scores revealed a mean (SD) pain severity of 5.3 (1.9). The weekly pain episodes lasted for 2.50 (8.3) hours.

### Phantom limb pain risk factors

**Fig. 2:**
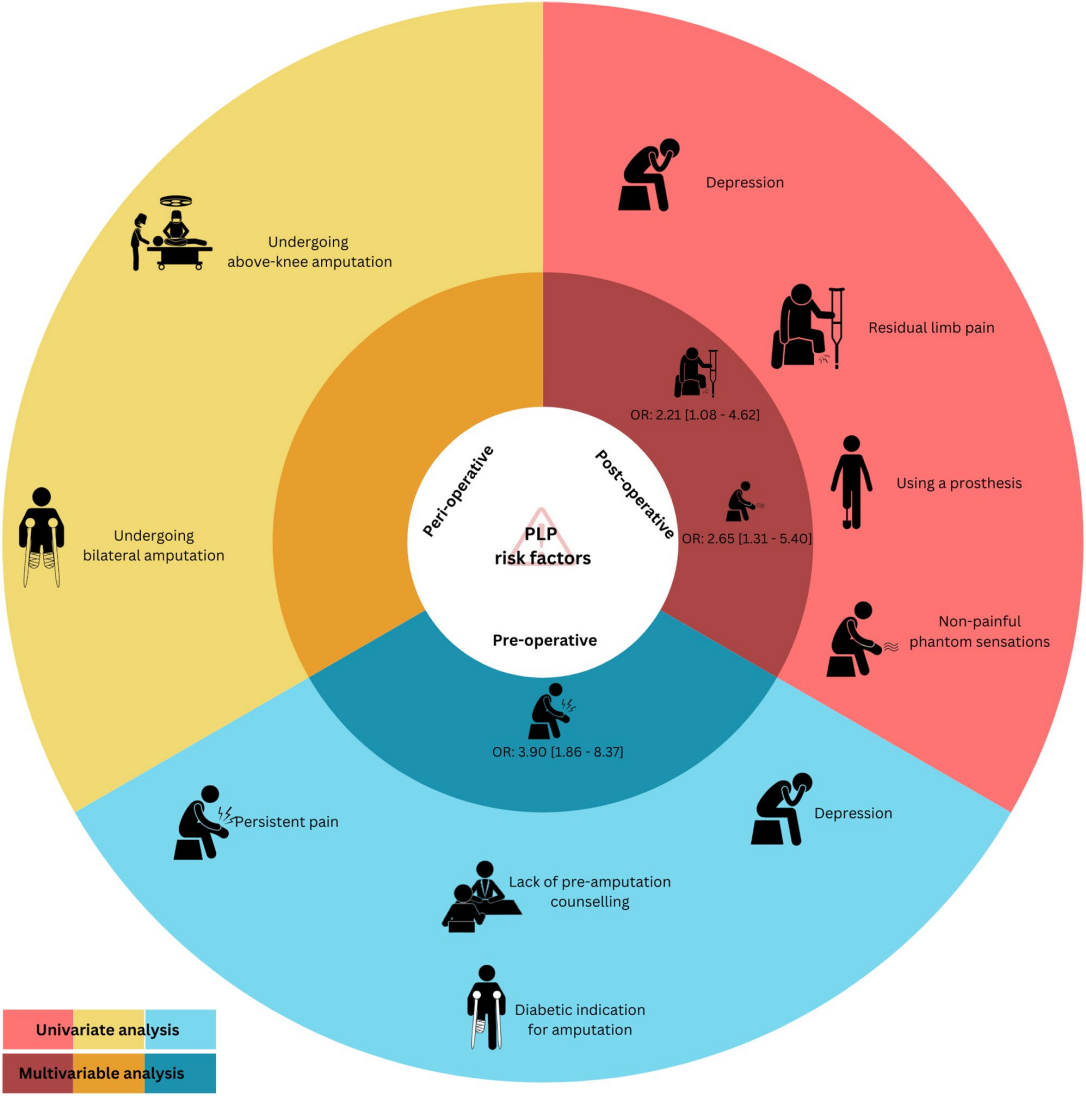
The univariate and multivariable analyses of risk factors for PLP (n = 231)

Phantom limb pain risk factors are presented in Fig. 2.

The univariate logistic regression analyses revealed associations between PLP and persistent pre-operative pain [OR 5.88 (3.04–11.6)], non-painful phantom limb sensations [OR 3.27 (1.78–6.05)], and residual limb pain [OR 4.05 (2.17–7.91)]. These associations were confirmed in the multivariable logistic regression analysis. The univariate logistic regression analysis revealed a negative association between PLP and undergoing pre-amputation counselling [OR 0.40 (0.20–0.77)], i.e., undergoing pre-amputation counselling reduced the risk of developing PLP. However, no firm association was confirmed in the multivariable logistic regression analysis [OR 1.72 (0.80–3.80)]. No associations were shown between PLP and other variables entered into our logistic regression model. Hosmer-Lemeshow statistic was χ^2^ = 7, *p* = 0.42. The high p-value fails to suggest that our logistic regression model is a poor fit [14].

## Discussion

This study aimed to evaluate the prevalence and characteristics of PLP and associated risk factors in South African people with LLAs. Our findings revealed a prevalence estimate of 71.73% (95% CI: 65.45–77.46) for PLP. Furthermore, this study identified persistent pre-operative pain, residual limb pain, and non-painful phantom limb sensations as important risk factors for PLP.

### PLP prevalence

Our study revealed a high prevalence of PLP, consistent with the findings of another Africa-based study [63.64% (95% CI: 53.92–72.60)] [15], but significantly higher than the PLP prevalence in Africans with traumatic Upper Limb Amputations (ULAs) [32.50% (95% CI: 18.57–49.13)] [16]. Higher PLP estimates are commonly observed in people with LLAs than in those with ULAs [17, 18]. The high PLP prevalence in people with LLAs may be explained by the role of risk factors (e.g., persistent pre-amputation pain due to diabetic neuropathy), which are typically not seen in people with ULAs, who are typically healthy and often undergo amputation due to trauma [19]. The high PLP prevalence and ‘worst pain’ scores suggest that many people with amputations are suffering from pain. Therefore, strategies including patient and healthcare provider education need to be implemented effectively to improve the patient-centred prevention and management of PLP in people with limb amputations.

It was not surprising that complications due to uncontrolled diabetes were the most common indication for amputations in this study. The International Diabetes Federation estimated that approximately 4.5 million people in South Africa had diabetes in 2019 [20]. Considering the steady rise in cases reported since 2009, the prevalence of diabetes is predicted to rise significantly in the future [2, 21]. This chronic disease of lifestyle largely affects people in low-income households, who are less educated about the condition and have difficulty maintaining healthy eating habits due to the perceived high cost of healthy food [22]. Low health literacy is associated with poor self-management strategies, deterioration in health status, increased chances of hospitalization, and several complications that may result in limb amputation surgery [23]. Altogether, these findings motivate for the design and implementation of evidence- and population-based education programmes focusing on chronic diseases of lifestyle, including diabetes, and strategies for prevention and self-management.

### Risk factors

Our findings showed that people with PLP were two times more likely to have experienced persistent pre-amputation pain than those without PLP. Studies conducted elsewhere indicate that people with PLP are three to ten times more likely to have experienced persistent pre-amputation pain [24, 25]. The strong association between pre-amputation pain and PLP may be explained by central sensitization – a physiological mechanism where persistent pain prior to amputation contributes to the hyperexcitability of the central nervous system, which may continue to upregulate nociceptive activity after amputation, thus resulting in PLP with similar characteristics. This causal mechanistic relationship between PLP and pre-amputation pain was first suggested by Jensen et al. [26] after revealing striking similarities in the characteristics (nature, quality, and severity) of pre-amputation pain and PLP. This idea has since been supported by other studies showing that over 60% of the people who experienced persistent pre-amputation pain experience PLP with similar characteristics [27, 28]. The strong association between pre-amputation pain and PLP also highlights the need to optimise perioperative pain management using multimodal analgesia, particularly in individuals with uncontrolled diabetes and limb infections, who may have experienced pre-amputation pain [29, 30].

Interestingly, our findings revealed that individuals with PLP were more likely to have experienced non-painful phantom limb sensations than those without pain. Other studies have reported similar findings, with 70–100% of amputees who reported non-painful phantom sensations also experiencing PLP [31, 32]. This co-occurrence suggests that non-painful sensations share neural mechanisms with PLP [48]. Studies have shown that inducing PLP and non-painful phantom sensations activate somatosensory and premotor cortices contralateral to the amputated limb [33, 34]. The similarities in cortical activation patterns might explain a connection between PLP and non-painful phantom sensations.

### Limitations

We could not recruit more participants beyond the attained sample size because we had exhausted the list of patients who had given consent to be contacted for research purposes. Therefore, this study is prone to selection bias, and its findings may not be generalizable to other population groups. This study used a cross-sectional design to evaluate risk factors for PLP. This design (compared to a cohort design) is subject to recall bias in that patients may not accurately recall their exposure to some risk factors prior to the onset of PLP. We recommend that future studies use a prospective longitudinal cohort design to provide robust results on important risk factors for PLP in the African population. The phrasing used in the questionnaire evaluating risk factors (Supplementary file 2) was not neutral, which may have led to a response bias. In particular, the use of the term ‘risk factors” may have biased responses and could be replaced in future studies with the term “influencing factors”. In addition, identifying a lack of pre-amputation counselling/support as increasing risk for phantom limb pain may also have biased responses. In future studies, this could be addressed by asking whether pre-amputation counselling/support was or was not received. Lastly, the impact of ethnicity and socio-cultural factors on PLP was not investigated in this study. We recommend that further studies explore the potential relationship between these factors and PLP in individuals with amputations.

## Conclusion

Our study indicates that roughly seven out of 10 people with LLAs experience PLP. This prevalence is high, and healthcare professionals ought to optimise peri-operative pain management to prevent post-surgical pain complications. In addition, healthcare professionals ought to implement early post-operative screening processes for PLP to effect timely pain management. The identification of persistent pre-amputation pain as a modifiable risk factor for PLP in this patient group may yield more effective and targeted pre-amputation care, leading to improved quality of life after amputation.

### Electronic supplementary material

Below is the link to the electronic supplementary material.

Supplementary Material 1

Supplementary Material 2

Supplementary Material 3

## Data Availability

All data reported in this manuscript will be made available upon reasonable request.
