# Supplementary material for: The prevalence and risk factors for phantom limb pain: a cross-sectional survey
Source: BMC Neurol. 2024 Feb 6;24:57. doi: 10.1186/s12883-024-03547-w (PMC10845739; doi:10.1186/s12883-024-03547-w)
Supplement: Supplementary file 1 — Supplementary Material 1 [file 12883_2024_3547_MOESM1_ESM.docx]

Supplementary file 2: Customised assessment sheet.

**A: Risk factors for phantom limb pain**

Pre-operative risk factors

Persistent pre-operative pain:

Did you experience continuous pain in the limb before it was amputated?

Yes No

Diabetic cause of amputation:

Was your limb amputated because of complications of diabetes?

Yes No

Traumatic cause of amputation:

Was your limb amputated because of an accident?

Yes No

Pre-amputation depression:

Were you diagnosed with depression before your limb was amputated?

Yes No

Pre-amputation counselling/support

Did you receive counselling about your amputation before your limb was amputated?

Yes No

Perioperative risk factors:

Proximal site of amputation:

Was your limb amputated above the elbow or knee?

Yes No

Lower limb amputation:

Was the amputation done on your leg?

Yes No

Bilateral amputation:

Were both your left and right limb amputated?

Yes No

Post-operative risk factors:

Depression:

Have you been diagnosed with depression since your limb was amputated?

Yes No

Stump pain:

Do you experience pain in the remaining part of the amputated limb?

Yes No

Use of Prosthesis:

Do you use a prosthetic limb?

Yes No

If so, please indicate the type of prosthesis you use: Mechanical Cosmetic Myoelectric

Phantom sensations:

Do you feel non-painful sensations in the limb that is no longer there?

Yes No

**B: Customised phantom limb pain questionnaire.**

Many people who have had amputations report that they can still feel their limb or have pain in the limb which is no longer there. Being able to feel the limb is called phantom limb sensation. Having pain in the limb which is no longer there is called phantom limb pain. In the past week, have you experienced phantom limb pain – pain in your limb which is no longer there?

Why was your limb amputated?

.

Phantom limb pain characteristics.

What words would you use to describe the pain you are experiencing in the limb which is no longer there?

Burning Sharp Shooting

Aching Cramping Other

Elaborate: .

Phantom limb pain episodes.

How many times in the past week have you experienced phantom limb pain?

Number of pain episodes: .

Phantom limb pain duration

How many minutes does each episode usually last?

Duration of episode: .
