# Supplementary material for: The prevalence and risk factors for phantom limb pain: a cross-sectional survey
Source: BMC Neurol. 2024 Feb 6;24:57. doi: 10.1186/s12883-024-03547-w (PMC10845739; doi:10.1186/s12883-024-03547-w)
Supplement: Supplementary file 2 — Supplementary Material 2 [file 12883_2024_3547_MOESM2_ESM.docx]

STROBE Statement—checklist of items that should be included in reports of observational studies.

|  | Item No. | Recommendation | Page  No. | Relevant text from manuscript |
| --- | --- | --- | --- | --- |
| **Title and abstract** | 1 | (*a*) Indicate the study’s design with a commonly used term in the title or the abstract | Page 1 | The prevalence and risk factors for phantom limb pain: a cross-sectional survey. |
|  |  | (*b*) Provide in the abstract an informative and balanced summary of what was done and what was found | Page 2 | We conducted a cross-sectional study using 231 amputation cases. Data on the prevalence and risk factors for PLP were collected telephonically from consenting and eligible participants. The overall PLP prevalence was 71.73% [95% CI: 65.45 – 77.46]. Persistent pre-operative pain, residual limb pain and non-painful phantom limb sensations were identified as risk factors for PLP. |
| Introduction | | | |  |
| Background/rationale | 2 | Explain the scientific background and rationale for the investigation being reported | Page 3 | Remarkably, the evidence is lacking on the prevalence of PLP and associated risk factors in African populations (1). Therefore, the burden of pain and potential targets for treatment in this patient group are not clearly understood. |
| Objectives | 3 | State specific objectives, including any prespecified hypotheses | Page 4 | Therefore, we conceived this study with the aim of exploring the prevalence of PLP and associated risk factors in South African people with Lower Limb Amputations (LLAs). |
| Methods | | | |  |
| Study design | 4 | Present key elements of study design early in the paper | Page 4 | We conducted a cross-sectional study with a convenience sample of people with LLAs. |
| Setting | 5 | Describe the setting, locations, and relevant dates, including periods of recruitment, exposure, follow-up, and data collection | Page 4 | The study was conducted at three tertiary healthcare facilities based in the Western Cape and Eastern Cape provinces between January 2018 and October 2022. |
| Participants | 6 | *Cross-sectional study*—Give the eligibility criteria, and the sources and methods of selection of participants | Page 5 | We included adults (≥18 years) who had undergone surgical or traumatic LLAs between January 2018 and October 2022, and were able to speak English, isiXhosa or Afrikaans languages. Participants were excluded if they had auditory or speech impairments in such a way that they were unable to hear or speak clearly via telephone. |
| Variables | 7 | Clearly define all outcomes, exposures, predictors, potential confounders, and effect modifiers. Give diagnostic criteria, if applicable | Page 5 | The primary outcome was PLP assessed using the pain severity scale of the Brief Pain Inventory (BPI). The secondary outcome was PLP risk factors assessed using a pre-piloted customised tool. |
| Data sources/ measurement | 8* | For each variable of interest, give sources of data and details of methods of assessment (measurement). Describe comparability of assessment methods if there is more than one group | Page 5 | The primary outcome was PLP assessed using the pain severity scale of the Brief Pain Inventory (BPI). The secondary outcome was PLP risk factors assessed using a pre-piloted customised tool. |
| Bias | 9 | Describe any efforts to address potential sources of bias | Page 14 | We had hoped to randomly select the required number of participants from the database of people with amputations, to minimise selection bias and to have sample that is representative of the amputee population. However, a small pool of patients in this database meant we had to use a convenience sample. |
| Study size | 10 | Explain how the study size was arrived at | Page 5 | The sample size was calculated using the formula [$n=\frac{Z^{2} P(1-P)}{d^{2}}$] developed by Daniel (11) for calculating a sample size in prevalence studies. |

Continued on next page

| Quantitative variables | 11 | Explain how quantitative variables were handled in the analyses. If applicable, describe which groupings were chosen and why | Page 6 | The prevalence of PLP was calculated by dividing the number of PLP cases with the number of amputation cases. For example, if the participant had two amputated limbs but had PLP in one of them, we recorded this as one PLP case and two amputation cases. The overall prevalence of PLP was expressed as a percentage with a 95% confidence interval. The association between risk factors and PLP were tested using univariate logistic regression analyses. Covariates associated with PLP at this stage were entered into the multivariable logistic regression model to examine the adjusted effects of the variables on the association between covariates and PLP (14). We excluded interrelated independent variables (e.g., pre-amputation depression and post-amputation depression) to increase the robustness of the multivariable model. |
| --- | --- | --- | --- | --- |
| Statistical methods | 12 | (*a*) Describe all statistical methods, including those used to control for confounding | Pages 6 – 7 | Data were analysed using ‘R’ version 4.2.2 - a statistical tool available on [www.R-project.org](http://www.R-project.org). The prevalence of PLP was calculated by dividing the number of PLP cases with the number of amputation cases. For example, if the participant had two amputated limbs but had PLP in one of them, we recorded this as one PLP case and two amputation cases. The overall prevalence of PLP was expressed as a percentage with a 95% confidence interval. The association between risk factors and PLP were tested using univariate logistic regression analyses. Covariates associated with PLP at this stage were entered into the multivariable logistic regression model to examine the adjusted effects of the variables on the association between covariates and PLP (14). We excluded interrelated independent variables (e.g., pre-amputation depression and post-amputation depression) to increase the robustness of the multivariable model. The associations between covariates and PLP were reported as Odds Ratio (OR) with a 95% confidence interval (15). The median and Inter-Quartile Range (IQR) were used to analyse numerical baseline data. Characteristics of PLP were reported descriptively. Statistical significance was set at p<0.05 for all analyses. |
|  |  | (*b*) Describe any methods used to examine subgroups and interactions |  | N/A |
|  |  | (*c*) Explain how missing data were addressed |  | N/A |
|  |  | (*d*) *Cohort study*—If applicable, explain how loss to follow-up was addressed  *Case-control study*—If applicable, explain how matching of cases and controls was addressed  *Cross-sectional study*—If applicable, describe analytical methods taking account of sampling strategy |  | N/A |
|  |  | (*e*) Describe any sensitivity analyses |  | N/A |
| Results | | | | |
| Participants | 13* | (a) Report numbers of individuals at each stage of study—e.g numbers potentially eligible, examined for eligibility, confirmed eligible, included in the study, completing follow-up, and analysed | Page 7 | The study included 208 participants [male (n=133); female (n=75)] with a mean (SD) age of 57.8 (12.8) (Table 1). Because 23 participants had undergone double amputations, the analysis was performed on a total of 231 cases. |
|  |  | (b) Give reasons for non-participation at each stage |  | N/A |
|  |  | (c) Consider use of a flow diagram | Page 7 | Figure 1 |
| Descriptive data | 14* | Give characteristics of study participants (eg demographic, clinical, social) and information on exposures and potential confounders | Page 7 | All the participants had LLAs, with most having amputations above the knee (57%). The participants had undergone amputation surgery for approximately 10 months prior to recruitment. The common indications for amputation were complications due to uncontrolled diabetes, infection, limb ischaemia, and cancer. The terms that were commonly used to describe the PLP were sharp, burning, and shooting (Table 2). |
| Outcome data | 15* | *Cross-sectional study—*Report numbers of outcome events or summary measures | Page 9 | The prevalence of PLP during the week preceding data collection was 71.73% [95% CI: 65.45 – 77.46]. The participants experienced a mean (SD) of 3.88 (2.34) PLP episodes per week, with a mean (SD) pain severity score of 2.19 (1.81). The weekly pain episodes lasted for 2.50 (8.3) hours. |
| Main results | 16 | (*a*) Give unadjusted estimates and, if applicable, confounder-adjusted estimates and their precision (eg, 95% confidence interval). Make clear which confounders were adjusted for and why they were included | Page 9 | The prevalence of PLP during the week preceding data collection was 71.73% [95% CI: 65.45 – 77.46]. The participants experienced a mean (SD) of 3.88 (2.34) PLP episodes per week, with a mean (SD) pain severity score of 2.19 (1.81). The weekly pain episodes lasted for 2.50 (8.3) hours. Phantom limb pain risk factors are presented in Table 3.  The univariate logistic regression analyses revealed associations between PLP and persistent pre-operative pain [OR 5.88 (3.04 – 11.6)], non-painful phantom limb sensations [OR 3.27 (1.78 – 6.05)], and residual limb pain [OR 4.05 (2.17 – 7.91)]. These associations were confirmed in the multivariable logistic regression analysis. The univariate logistic regression analysis revealed a negative association between PLP and undergoing pre-amputation counselling [OR 0.40 (0.20 – 0.77)], i.e., undergoing pre-amputation counselling reduced the risk of developing PLP. However, no firm association was confirmed in the multivariable logistic regression analysis [OR 1.72 (0.80 – 3.80)]. No associations were shown between PLP and other variables. |

Continued on next page

| Other analyses | 17 | Report other analyses done—eg analyses of subgroups and interactions, and sensitivity analyses |  | N/A |
| --- | --- | --- | --- | --- |
| Discussion | | | | |
| Key results | 18 | Summarise key results with reference to study objectives | Page 12 | The aim of the study was to evaluate the prevalence and characteristics of PLP and associated risk factors in South African people with limb amputations. Our findings revealed a prevalence estimate of 71.73% (95% CI: 65.45 – 77.46) for PLP. Furthermore, this study identified persistent pre-operative pain, residual limb pain, and non-painful phantom limb sensations as important risk factors for PLP. |
| Limitations | 19 | Discuss limitations of the study, taking into account sources of potential bias or imprecision. Discuss both direction and magnitude of any potential bias | Page 13 | We could not recruit more participants beyond the attained sample size because we had exhausted the list of patients who had given consent to be contacted for research purposes. Therefore, this study is prone to selection bias, and its findings may not be generalizable to other population groups. This study used a cross-sectional design to evaluate risk factors for PLP. This design (compared to a cohort design) is subject to recall bias in that patients may not accurately recall their exposure to some risk factors prior to the onset of PLP. We recommend that future studies use a prospective longitudinal cohort design to provide robust results on important risk factors for PLP in the African population. The phrasing used in the questionnaire evaluating risk factors (Supplementary file 2) was not neutral, which may have led to a response bias. In particular, the use of the term 'risk factors" may have biased responses and could be replaced in future studies with the term "influencing factors". In addition, identifying a lack of pre-amputation counselling/support as increasing risk for phantom limb pain may also have biased responses. In future studies, this could be addressed by asking whether pre-amputation counselling/support was or was not received. Lastly, the impact of ethnicity and socio-cultural factors on PLP was not investigated in this study. We recommend that further studies explore the potential relationship between these factors and PLP in individuals with amputations. |
| Interpretation | 20 | Give a cautious overall interpretation of results considering objectives, limitations, multiplicity of analyses, results from similar studies, and other relevant evidence | Page 13 | Our study indicates that roughly seven out of 10 people with LLAs experience PLP. This prevalence is high, and healthcare professionals ought to optimise peri-operative pain management to prevent post-surgical pain complications. In addition, healthcare professionals ought to implement effective post-operative screening processes for PLP to effect timely pain management. The identification of persistent pre-amputation pain as a modifiable risk factor for PLP in this patient-group may yield more effective and targeted pre-amputation care, leading to improved quality of life after amputation. |
| Generalisability | 21 | Discuss the generalisability (external validity) of the study results |  | N/A |
| Other information | |  | | |
| Funding | 22 | Give the source of funding and the role of the funders for the present study and, if applicable, for the original study on which the present article is based | Page 15 | K.L. was awarded the Start-up Emerging Researcher grant by the University of Cape Town. |

*Give information separately for cases and controls in case-control studies and, if applicable, for exposed and unexposed groups in cohort and cross-sectional studies.

**Note:** An Explanation and Elaboration article discusses each checklist item and gives methodological background and published examples of transparent reporting. The STROBE checklist is best used in conjunction with this article (freely available on the Web sites of PLoS Medicine at http://www.plosmedicine.org/, Annals of Internal Medicine at http://www.annals.org/, and Epidemiology at http://www.epidem.com/). Information on the STROBE Initiative is available at www.strobe-statement.org.
