# Supplementary material for: The prevalence and risk factors for phantom limb pain: a cross-sectional survey
Source: BMC Neurol. 2024 Feb 6;24:57. doi: 10.1186/s12883-024-03547-w (PMC10845739; doi:10.1186/s12883-024-03547-w)
Supplement: Supplementary file 3 — Supplementary Material 3 [file 12883_2024_3547_MOESM3_ESM.html]

Analysis-1.R


# Analysis-1.R

#### RaymondNhapi

#### 2022-11-11

```
setwd("~/KL") # set working directory

rm(list = ls()) # clears all that's in R memory at the time


# The libraries are packages of functions
# - you load a library that you want to use for the session...
library(readxl) # reading excel files
library(epiR) # for prevalences
```

```
## Warning: package 'epiR' was built under R version 4.2.2
```

```
## Loading required package: survival
```

```
## Package epiR 2.0.53 is loaded
```

```
## Type help(epi.about) for summary information
```

```
## Type browseVignettes(package = 'epiR') to learn how to use epiR for applied epidemiological analyses
```

```
##
```

```
library(dplyr) # data manipulation & summaries
```

```
## 
## Attaching package: 'dplyr'
```

```
## The following objects are masked from 'package:stats':
## 
##     filter, lag
```

```
## The following objects are masked from 'package:base':
## 
##     intersect, setdiff, setequal, union
```

```
library(broom) # for odds ratios

# reading the data into R
pilot_data <- read_excel("Analyses_PLP.xlsx", sheet = "Sheet1")

# data summaries
pilot_data %>% summarise(mean(Age),
                         sd(Age))
```

```
## # A tibble: 1 × 2
##   `mean(Age)` `sd(Age)`
##         <dbl>     <dbl>
## 1        57.8      12.8
```

```
pilot_data %>% summarise(mean(`Months since amputation`),
                         sd(`Months since amputation`))
```

```
## # A tibble: 1 × 2
##   `mean(\`Months since amputation\`)` `sd(\`Months since amputation\`)`
##                                 <dbl>                             <dbl>
## 1                                9.76                              9.32
```

```
pilot_data %>% summarise(mean(`Pain severity score`),
                         sd(`Pain severity score`),
                         min(`Pain severity score`))
```

```
## # A tibble: 1 × 3
##   `mean(\`Pain severity score\`)` `sd(\`Pain severity score\`)` min(\Pain seve…¹
##                             <dbl>                         <dbl>            <dbl>
## 1                            2.19                          1.81                0
## # … with abbreviated variable name ¹​`min(\`Pain severity score\`)`
```

```
# by subgroup: M/F
pilot_data %>% # take my data
  group_by(Sex) %>% # group the data
  summarise(mean(Age),sd(Age)) # summarise some stuff in the groups
```

```
## # A tibble: 2 × 3
##   Sex    `mean(Age)` `sd(Age)`
##   <chr>        <dbl>     <dbl>
## 1 female        59.9      10.9
## 2 male          56.5      13.6
```

```
# by subgroup: PLP
pilot_data %>% # take my data
  filter(PLP=="Y") %>% 
  summarise(mean(`Pain severity score`),
            sd(`Pain severity score`)) # summarise some stuff in the groups
```

```
## # A tibble: 1 × 2
##   `mean(\`Pain severity score\`)` `sd(\`Pain severity score\`)`
##                             <dbl>                         <dbl>
## 1                            3.05                          1.38
```

```
pilot_data %>% # take my data
  filter(PLP=="Y") %>% 
  summarise(mean(`Episodes/week`),
            sd(`Episodes/week`)) # summarise some stuff in the groups
```

```
## # A tibble: 1 × 2
##   `mean(\`Episodes/week\`)` `sd(\`Episodes/week\`)`
##                       <dbl>                   <dbl>
## 1                      3.88                    2.34
```

```
pilot_data %>% # take my data
  filter(PLP=="Y") %>% 
  summarise(mean(`Duration of episodes(min)/week`),
            sd(`Duration of episodes(min)/week`)) # summarise some stuff in the groups
```

```
## # A tibble: 1 × 2
##   `mean(\`Duration of episodes(min)/week\`)` sd(\Duration of episodes(min)/wee…¹
##                                        <dbl>                               <dbl>
## 1                                       149.                                496.
## # … with abbreviated variable name ¹​`sd(\`Duration of episodes(min)/week\`)`
```

```
pilot_data %>% # take my data
  filter(PLP=="Y") %>% 
  mutate(`Duration of episodes(hrs)/week`= `Duration of episodes(min)/week`/60) %>%
  summarise(mean(`Duration of episodes(hrs)/week`),
            sd(`Duration of episodes(hrs)/week`)) # summarise some stuff in the groups
```

```
## # A tibble: 1 × 2
##   `mean(\`Duration of episodes(hrs)/week\`)` sd(\Duration of episodes(hrs)/wee…¹
##                                        <dbl>                               <dbl>
## 1                                       2.48                                8.26
## # … with abbreviated variable name ¹​`sd(\`Duration of episodes(hrs)/week\`)`
```

```
library(ggplot2)
pilot_data %>% # take my data
  filter(PLP=="Y") %>% 
  mutate(`Duration of episodes(hrs)/week`= `Duration of episodes(min)/week`/60) %>%
  ggplot(aes(x = `Duration of episodes(hrs)/week`)) +geom_histogram()
```

```
## `stat_bin()` using `bins = 30`. Pick better value with `binwidth`.
```

```
# Computing prevalences
ncas <- sum(pilot_data$PLP == "Y") # number of cases
npop <- nrow(pilot_data) # total number of subjects
tmp <- as.matrix(cbind(ncas, npop))
epi.conf(tmp, ctype = "prevalence", method = "exact",  design = 1, 
         conf.level = 0.95) * 100
```

```
##        est    lower    upper
## 1 71.73913 65.44709 77.46115
```

```
# Computing prevalences by group( `Months since amputation`)
amputation_less_12m <- pilot_data %>% filter(`Months since amputation`<12)
amputation_more_12m <- pilot_data %>% filter(`Months since amputation`>=12)

# for less than 12m
ncas <- sum(amputation_less_12m$`PLP` == "Y") # number of cases
npop <- nrow(amputation_less_12m) # total number of subjects
tmp <- as.matrix(cbind(ncas, npop))
epi.conf(tmp, ctype = "prevalence", method = "exact",  design = 1, 
         conf.level = 0.95) * 100
```

```
##        est    lower    upper
## 1 83.22581 76.39596 88.74026
```

```
# for more than 18m
ncas <- sum(amputation_more_12m$`PLP` == "Y") # number of cases
npop <- nrow(amputation_more_12m) # total number of subjects
tmp <- as.matrix(cbind(ncas, npop))
epi.conf(tmp, ctype = "prevalence", method = "exact",  design = 1, 
         conf.level = 0.95) * 100
```

```
##   est    lower    upper
## 1  48 36.31353 59.84924
```

```
# logistic regression
m_Sex <- glm(as.factor(PLP)~Sex,
             data = pilot_data,
             family = binomial(link = "logit"))
#summary(m_Sex) # estimates given on the logit scale
broom::tidy(m_Sex, conf.int = TRUE) %>%
  mutate(OR = exp(estimate),conf.low = exp(conf.low), conf.high=exp(conf.high))%>% 
  dplyr::select(term,OR, conf.low,conf.high)%>%
  filter(term!="(Intercept)")
```

```
## # A tibble: 1 × 4
##   term       OR conf.low conf.high
##   <chr>   <dbl>    <dbl>     <dbl>
## 1 Sexmale 0.620    0.328      1.14
```

```
# Diabetic
m_cause <- glm(as.factor(PLP)~I(Cause=="Diabetic complications"),
             data = pilot_data,
             family = binomial(link = "logit"))
broom::tidy(m_cause, conf.int = TRUE) %>%
  mutate(OR = exp(estimate),conf.low = exp(conf.low), conf.high=exp(conf.high))%>% 
  dplyr::select(term,OR, conf.low,conf.high)%>%
  filter(term!="(Intercept)")
```

```
## # A tibble: 1 × 4
##   term                                            OR conf.low conf.high
##   <chr>                                        <dbl>    <dbl>     <dbl>
## 1 "I(Cause == \"Diabetic complications\")TRUE" 0.750    0.420      1.33
```

```
# Prep-op depression
m_cause <- glm(as.factor(PLP)~`Pre-op Depression`,
               data = pilot_data,
               family = binomial(link = "logit"))
broom::tidy(m_cause, conf.int = TRUE) %>%
  mutate(OR = exp(estimate),conf.low = exp(conf.low), conf.high=exp(conf.high))%>% 
  dplyr::select(term,OR, conf.low,conf.high) %>%
  filter(term!="(Intercept)")
```

```
## # A tibble: 1 × 4
##   term                    OR conf.low conf.high
##   <chr>                <dbl>    <dbl>     <dbl>
## 1 `Pre-op Depression`Y 0.552    0.258      1.21
```

```
# Prep-op counselling
m_cause <- glm(as.factor(PLP)~I(`Pre-op counseling`=="N"),
               data = pilot_data,
               family = binomial(link = "logit"))
broom::tidy(m_cause, conf.int = TRUE) %>%
  mutate(OR = exp(estimate),conf.low = exp(conf.low), conf.high=exp(conf.high))%>% 
  dplyr::select(term,OR, conf.low,conf.high) %>%
  filter(term!="(Intercept)")
```

```
## # A tibble: 1 × 4
##   term                                     OR conf.low conf.high
##   <chr>                                 <dbl>    <dbl>     <dbl>
## 1 "I(`Pre-op counseling` == \"N\")TRUE" 0.402    0.200     0.766
```

```
# Bilateral amputation
m_cause <- glm(as.factor(PLP)~`Bilateral amputation`,
               data = pilot_data,
               family = binomial(link = "logit"))
broom::tidy(m_cause, conf.int = TRUE) %>%
  mutate(OR = exp(estimate),conf.low = exp(conf.low), conf.high=exp(conf.high))%>% 
  dplyr::select(term,OR, conf.low,conf.high) %>%
  filter(term!="(Intercept)")
```

```
## # A tibble: 1 × 4
##   term                       OR conf.low conf.high
##   <chr>                   <dbl>    <dbl>     <dbl>
## 1 `Bilateral amputation`Y 0.528    0.283     0.994
```

```
# Post-op depression
m_cause <- glm(as.factor(PLP)~`Post-op depression`,
               data = pilot_data,
               family = binomial(link = "logit"))
broom::tidy(m_cause, conf.int = TRUE) %>%
  mutate(OR = exp(estimate),conf.low = exp(conf.low), conf.high=exp(conf.high))%>% 
  dplyr::select(term,OR, conf.low,conf.high) %>%
  filter(term!="(Intercept)")
```

```
## # A tibble: 1 × 4
##   term                     OR conf.low conf.high
##   <chr>                 <dbl>    <dbl>     <dbl>
## 1 `Post-op depression`Y  1.14    0.478      3.04
```

```
# Stump pain
m_cause <- glm(as.factor(PLP)~`Stump pain`,
               data = pilot_data,
               family = binomial(link = "logit"))
broom::tidy(m_cause, conf.int = TRUE) %>%
  mutate(OR = exp(estimate),conf.low = exp(conf.low), conf.high=exp(conf.high))%>% 
  dplyr::select(term,OR, conf.low,conf.high) %>%
  filter(term!="(Intercept)")
```

```
## # A tibble: 1 × 4
##   term             OR conf.low conf.high
##   <chr>         <dbl>    <dbl>     <dbl>
## 1 `Stump pain`Y  4.05     2.17      7.91
```

```
# prosthesis use
m_cause <- glm(as.factor(PLP)~`Prosthesis use`,
               data = pilot_data,
               family = binomial(link = "logit"))
broom::tidy(m_cause, conf.int = TRUE) %>%
  mutate(OR = exp(estimate),conf.low = exp(conf.low), conf.high=exp(conf.high))%>% 
  dplyr::select(term,OR, conf.low,conf.high) %>%
  filter(term!="(Intercept)")
```

```
## # A tibble: 1 × 4
##   term                 OR conf.low conf.high
##   <chr>             <dbl>    <dbl>     <dbl>
## 1 `Prosthesis use`Y 0.222   0.0445     0.933
```

```
# `phantom limb sensation`
m_cause <- glm(as.factor(PLP)~`phantom limb sensation`,
               data = pilot_data,
               family = binomial(link = "logit"))
broom::tidy(m_cause, conf.int = TRUE) %>%
  mutate(OR = exp(estimate),conf.low = exp(conf.low), conf.high=exp(conf.high))%>% 
  dplyr::select(term,OR, conf.low,conf.high) %>%
  filter(term!="(Intercept)")
```

```
## # A tibble: 1 × 4
##   term                         OR conf.low conf.high
##   <chr>                     <dbl>    <dbl>     <dbl>
## 1 `phantom limb sensation`Y  3.27     1.78      6.05
```

```
# `Proximal site of amputation`
m_cause <- glm(as.factor(PLP)~`Proximal site of amputation`,
               data = pilot_data,
               family = binomial(link = "logit"))
broom::tidy(m_cause, conf.int = TRUE) %>%
  mutate(OR = exp(estimate),conf.low = exp(conf.low), conf.high=exp(conf.high))%>% 
  dplyr::select(term,OR, conf.low,conf.high) %>%
  filter(term!="(Intercept)")
```

```
## # A tibble: 1 × 4
##   term                              OR conf.low conf.high
##   <chr>                          <dbl>    <dbl>     <dbl>
## 1 `Proximal site of amputation`Y  1.42    0.796      2.53
```

```
# `Pre-op Pain`
m_cause <- glm(as.factor(PLP)~`Pre-op Pain`,
               data = pilot_data,
               family = binomial(link = "logit"))
broom::tidy(m_cause, conf.int = TRUE) %>%
  mutate(OR = exp(estimate),conf.low = exp(conf.low), conf.high=exp(conf.high))%>% 
  dplyr::select(term,OR, conf.low,conf.high) %>%
  filter(term!="(Intercept)")
```

```
## # A tibble: 1 × 4
##   term              OR conf.low conf.high
##   <chr>          <dbl>    <dbl>     <dbl>
## 1 `Pre-op Pain`Y  5.88     3.04      11.6
```

```
# multivariate
m_cause <- glm(as.factor(PLP)~Sex + 
                 `Pre-op Pain`+
                 `Diabetic cause of amputation` +
                 `Pre-op Depression`+
                 `Pre-op counseling`+
                 `Stump pain`+
                 `phantom limb sensation`+
                 `Proximal site of amputation`,
               data = pilot_data,
               family = binomial(link = "logit"))
broom::tidy(m_cause, conf.int = TRUE) %>%
  mutate(OR = exp(estimate),conf.low = exp(conf.low), conf.high=exp(conf.high))%>% 
  dplyr::select(term,OR, conf.low,conf.high) #%>%
```

```
## # A tibble: 9 × 4
##   term                               OR conf.low conf.high
##   <chr>                           <dbl>    <dbl>     <dbl>
## 1 (Intercept)                     0.745    0.227      2.40
## 2 Sexmale                         0.524    0.251      1.06
## 3 `Pre-op Pain`Y                  3.90     1.86       8.37
## 4 `Diabetic cause of amputation`Y 0.540    0.246      1.15
## 5 `Pre-op Depression`Y            0.425    0.172      1.06
## 6 `Pre-op counseling`Y            1.72     0.800      3.80
## 7 `Stump pain`Y                   2.21     1.08       4.62
## 8 `phantom limb sensation`Y       2.65     1.31       5.40
## 9 `Proximal site of amputation`Y  1.19     0.593      2.40
```

```
#  filter(term!="(Intercept)")
```
